# Quantification of urban mitigation potentials - coping with data heterogeneity

**DOI:** 10.1016/j.heliyon.2023.e16733

**Published:** 2023-05-30

**Authors:** Fabian Reitemeyer, David Fritz, Nikolai Jacobi, León Díaz-Bone, Carla Mariño Viteri, Juergen P. Kropp

**Affiliations:** a Potsdam Institute for Climate Impact Research – PIK, Member of Leibniz Association, P.O. Box 601203, Potsdam, 14412, Germany; b Environment Agency Austria, Spittelauer Lände 5, Vienna, 1090, Austria; c ICLEI European Secretariat, Leopoldring 3, Freiburg, 79098, Germany; d ICLEI - Local Governments for Sustainability e.V., Kaiser-Friedrich-Str. 7, Bonn, 53113, Germany; e Bauhaus Earth, Dortustraße 46, Potsdam, 14467, Germany; f University of Potsdam, Institute of Environmental Science and Geography, Karl-Liebknecht-Str. 24-25, Potsdam, 14476, Germany

**Keywords:** 0000, 1111, Mitigation potentials, Data heterogeneity, Sustainable building, Transport, Redensification

## Abstract

Cities are at the forefront of European and international climate action. However, in many cities, the ever-growing urban population is putting pressure on settlement and infrastructure development, increasing attention to urban planning, infrastructure and buildings. This paper introduces a set of quantification approaches, capturing impacts of urban planning measures in three fields of action: sustainable building, transport and redensification. The quantification approaches have been developed to account for different levels of data availability, thus providing users with quantification approaches that are applicable across cities. The mitigation potentials of various measures such as a modal shift, the substitution of building materials with wood, and different redensification scenarios were calculated. The substitution of conventional building materials with wood was analyzed as having a high mitigation potential. Building construction, in combination with urban planning and design, are key drivers for mitigating climate change in cities. Given the data heterogeneity among cities, mixed quantification approaches could be defined and the measures and policy areas with the greatest climate mitigation potential identified.

## 1 Introduction

Cities are at the forefront of European and international climate action. 55% of the global population live in urban areas and cities are responsible for an estimated 60% of global material processing or consumption and 70% of greenhouse gas (GHG) emissions [1]. Cities play an important role in achieving global sustainability as it is increasingly recognized at European level and in the context of international policy processes. Since 2015, this has been marked with a dedicated UN Sustainable Development Goal (SDG) on “Sustainable Cities and Communities” (SDG 11) [2]. More than 1000 cities have joined the UNFCCC's Cities Race to Zero, pledging actions to cut greenhouse gas (GHG) emissions to net zero by 2050, and to achieve ambitious interim targets by 2030 that are in line with the Paris Agreement [3].

In many countries the ever-increasing urban population puts pressure on settlement and infrastructure development in cities, which increases attention on urban planning, infrastructure and buildings - including their embedded and operational climate impacts (all the more imperative).

As the demand for physical infrastructure, especially buildings, is expected to double by 2050 [4], the building sector is emerging as one of the main sources of greenhouse gas emissions: which is annually responsible for 36% of global energy consumption and 37% of energy-related carbon dioxide emissions [5]. After clean water, concrete is the second most consumed product on earth [6]. The numbers vary widely, i.e., in Europe the building stock is responsible for the consumption of 33% of raw materials and 50% of electricity [7]. The building sector in the United States is accountable for 40% of energy consumption [8], while in China, the building sector accounts for 18% of total national GHG emissions [9].

Concrete is expected to contribute 12% of global GHG emissions by 2060 [10]. In 2050, the building sector globally needs to emit 80-90 per cent less GHG than in 2010, to remain compatible with the 1.5-degree target [11]. Against this backdrop, reducing the embodied carbon of buildings is being recognised as a key global area for action moving forward [12] and is the sector in which, for example, European cities define the most urban actions [13]. Churkina et al. (2020) suggests using sustainable timber for the construction of houses in order to store CO_2_ and make cities carbon-neutral. Himes and Busby showed that 9% global emissions can be reduced annually to meet the 2030 climate targets to stay below 1.5 degrees of warming if half of the expected new urban construction substitutes conventional materials with wood [14].

Emissions in the transport sector are rising faster than in all other sectors [15]. Emissions account for around a quarter of global emissions, with projections showing a doubling by 2050 [16]. The increase in GDP led to an increase in greenhouse gas emissions driven by its correlation with transport activities [17]. The slower growth rates or a decline in the use of private motorised transport in some cities in the global North, in turn, suggests a decoupling of this correlation [16]. In the global South in particular, rapid urbanisation and rising car ownership, especially in China and South East Asia, will double the number of light duty vehicles by mid-century [18]. Various levers exist to reduce emissions from transport, including improvements in transport technologies such as cars, planes and ships, economic instruments such as carbon taxes or fuel prices or behavioural changes such as the modal shift to climate-friendly transport modes [19]. Studies point to the importance of developing climate-oriented infrastructures in countries with rapid urbanisation, which will be crucial for the future emission intensity of transport and require land use measures to avoid gridlock [20], [21].

One of the most referenced strategies to contain additional land consumption from increased urbanization within paradigms of climate-friendly urban development is the compact city approach [22]. This urban development model anticipates GHG savings to occur through the densification of urban infrastructure, implying a more compact development and shorter travel distances, among other benefits [23]. Gudipudi et al. (2016) have shown that the density of urban settlements is negatively linearly correlated with emissions. In principle, this implies that further densification of settlement areas in lieu of expansion would result under constant population and gross domestic product (GDP) growth in considerable emission savings due to the improved energy utilization of multi-storey buildings and shorter travel distances. However, more compact urban development can incur trade-offs with regard to health, the urban heat island effect and the quality of life of inhabitants [24], [25]. Furthermore, it should be noted that focusing on the concentration of infrastructure alone does not address the spatial structure (e.g., polycentrism), which is decisive for the GHG mitigation potential across diverse fields of action [26]. In this light, the idea of the “15 minutes city”, which favours polycentric urban planning, could gain further relevance [27].

Urban areas in the Global South face the double challenge of having to address the dramatic consequences of climate change, by which they are disproportionally more affected, while managing high population growth rates and linked rapid urbanisation [28]. It is estimated that 90% of the 2.2 billion additional inhabitants that will be added to the global population by 2050 will reside in Africa and Asia [29]. It will therefore be of critical importance to ensure that cities in the Global South are enable to optimally exploit their GHG mitigation potentials by managing their growth via effective urban planning, whilst simultaneously dealing with financial and human resource limitations, technical capacity shortfalls, as well as informality [30].

This paper presents quantification approaches adapted to the heterogeneous data availability, capturing impacts of urban planning measures in the three fields of action: urban transport infrastructure, densification and building materials. The quantification approaches described in this paper have been developed and tested for different case - study cities across the global north & south to accommodate different levels of data availability. Taking into account this diversity, we provide a range of mixed bottom-up and top-down approaches for handling data heterogeneity. In doing so, cities will be provided with applicable ways of dealing with data heterogeneity to strengthen their mitigation pathways. Thereby contributing to the advancement of the Global Research and Action Agenda on Cities and Climate Change Science to be developed in the context of the IPCC's seventh assessment cycle, by providing insights into two of the outlined topical knowledge gaps on cities and climate change, namely urban planning and design as well as sustainable consumption and production. The following paper is organised as follows: first, in the methodological section, the case-study cities and the approaches used are presented. This is followed by a description of the different approaches adapted to the data heterogeneities. Following this, the results and a discussion with limitations are presented. At the end of the paper, a conclusion is given.

## 2 Methods

### 2.1 Case study cities

One of the key objectives underpinning this research was the validation of the selected measures, against the backdrop of data heterogeneity and socio-economic diversity. To explore the heterogeneity of dynamic urban development and data availability, therefore six cities with different characteristic phases of urban development and growth rates were selected. The cities were the regions where different approaches were used, and rather than with the focus on comparability. Among them cities with stable population growth and established administration, such as the cities of Leipzig and Essen (Germany). Over decades, a cohesive infrastructure has developed, with a focus on renovating old infrastructure and increasing resource efficiency. Nagpur and Rajkot (India) are examples of rapidly developing cities with infrastructural inequalities and a high proportion of informal settlements. The cities of Santa Rosa and Pasig City (Philippines), on the other hand, are examples of fast-growing cities that are redeveloping.

### 2.2 Selection of urban planning measures

**Figure 1:**
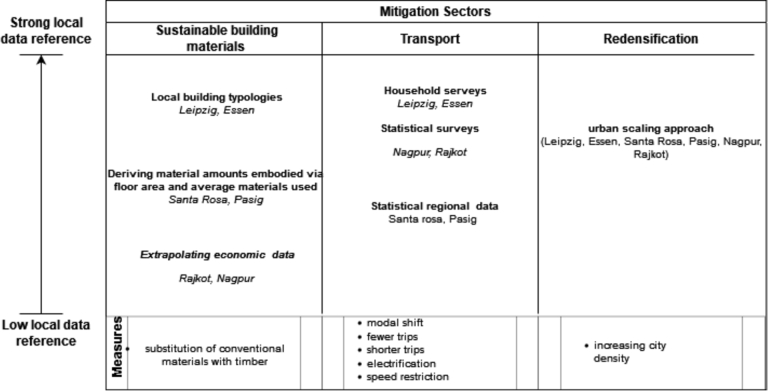
Visualisation of the different levels of data availability in the cities selected for closer examination per field of action.

Fig. 1 visualises the different levels of data availability in the cities selected for closer examination (sample city selection described in section 2.2) per field of action. In order to obtain an overview of the mitigation potentials that is as realistic as possible for the case - study cities, we adapted to the existing data heterogeneity and applied approaches with different spatial resolutions per field of action 1.

For the selection of mitigation measures, global datasets on reported measures to the CDP-ICLEI Track with expected GHG emission savings from cities were used [31]. These measures were then categorised into action fields and sorted by popularity among cities and amount of expected emission savings.

We chose to focus on overall mitigation across modes because discussions are focused on multimodal measures [32]. Mitigation potential that targets specific modes is targeted in other measures such as the modal shift from cars to non-motorised transportation.

An attempt was made to select measures that have a direct quantifable impact. For example, the impact of the measure of a 10% modal shift to non-motorised transport is direct quantifiable, in contrast to the measure of a general compact city approach (Table 1). The aim was to identify the most appropriate measures for each city in terms of GHG savings in each of the 3 action fields.

**Table 1:** Selected measures per action field and the magnitude of the average impact.

Mitigation measures	Estimation of the magnitude of the average impact
**Substitution of building materials**	
Focus on wood: 5% and 15%	Aligned with [33] and assumptions
**Transport**	
Modal shift to NMT (non motorised transport) 10% from Cars to NMT	[31]
Fewer trips 10% fewer trips on average	[31] and assumptions
Lower average trip length 35% lower trip length on average, not applied to NMT	[31], [34]
Efficiency gain in fleet 2% lower GHG emission factor	[35]
Speed restriction 25% lower max. speed in city border, leads to fewer pkm by 2.5%	[35]
Electrification of 50% of public bus fleet	aligned with national targets, [36]
50% electrification of 2- and 3-wheelers	aligned with national targets, [36]

**Redensification**	
Scenario 1: no changes in population and area	
Scenario 2: increase in population and area	[31] and specific urban population growth
Scenario 3: increase in population and no changes in area	

Models were then developed for each field of action, to quantify the potential emission savings. The models were based on the availability of data in the cities and were designed flexibly accordingly. The GHG saving per measure is always given in absolute terms and in % compared to the actual situation. Fig. 1 visualises the different levels of data availability in the cities selected for closer examination (sample city selection described in section 2.2) per field of action.

The approach presents a ‘what-if analysis’, assuming a thoroughly implementation of the separate selected measures.

### 2.3 Quantifying the impact of the selected measures

#### 2.3.1 Sustainable building and materials

The GHG emissions of the embodied building stock per city were calculated on the basis of the material stock estimates and by using suitable GHG emission factors (see SI). Subsequently, two scenarios were used to calculate the GHG savings through replacing abiotic parts of the building materials with an increased usage of timber. We chose replacement rates of abiotic material use is 5% and 15% respectively. For this, we used [33] and [37] as a reference, but in order to show low inhibition thresholds and accordingly more realistic urban pathways, the substitution rates are lower. Due to the different levels of data availability, three different approaches were employed (one per country).

The key difference between the three approaches is grounded in the modelling of construction materials for the building stock, while emission factors, correction factors as well as assumptions (measures) for substitution were applied across approaches and sample cities. Thus, whilst approaches differed to some extent, we ensured that these still allowed for comparison and validation of GHG savings and the approaches themselves.

**Modelling of building stock via building typologies (Germany)**

The most local data were available for the German cities, so this approach follows a bottom-up approach. For the German cities of Essen and Leipzig, the residential buildings were modelled using building cadastre data comprising the number of buildings constructed by decade and the type of use (multi-family or single-family house) [38], [39]. The total gross floor area per building age group was first calculated, based on the number of buildings and the average gross floor area. Standards and publications from city departments for the material composition of each building type were then applied. Here, the IÖR-information system “Gebaute Umwelt” (built environment) proved to be a valuable information resource [40]. The material consumption figures for buildings included in IÖR, are based on data for a number of typical representative buildings per age group, which are then aggregated [41]. The system also includes area/volume and material parameters (concrete, bricks, other mineral, sawn/processed wood, other renewable, plastics, ferrous metals and bitumen). The information system distinguishes between multi-family houses in West and East Germany from 1949-1990 (no differences between single family houses). In East Germany (applies to Leipzig), the buildings were mainly built-in prefabricated slab construction, with different materials and with more storeys.

The total quantity [t] of building materials was then calculated with the total gross floor area [m^2^] and the material input [t/m^2^] to obtain the total material input of the building stock for each city. Following this, the selected scenarios were applied to calculate the GHG saving through the use of replacement of parts of the building materials with timber (5% and 15%). In order to estimate GHG emissions, the building material quantities were calculated with correction and emission factors for timber (see SI).

**Extrapolating economic and material-flow data (India)**

Detailed data on the housing stock as well as material Flow Analysis (MFA) data on construction for the two Indian sample cities Nagpur and Rajkot was lacking. Therefore, using a comprehensive material flow study for six Asian cities from the Asian Development Bank (2014), the Domestic Material Input (DMI) indicator was derived for construction materials (cement, sand, gravel, steel, wood, plastics) for the city of Bangalore for the year 2000. Dividing Bangalore's total construction DMI by local GDP, the construction material intensity of local GDP can be calculated. The carbon intensity of construction materials was derived using the same global emission factors as in the other sample cities of this study (SI). A reason we opted to use global emission factors was to facilitate the replication of the GHG mitigation calculation approach in other cities and to enable comparison of quantification approaches for the construction sector. Furthermore, in order to ensure robustness of the extrapolation, another MFA data set (DMI_con_) with the associated emissions (Emissions_con_) was used from the City of Mumbai [42]. As was done for Bangalore, we derived the carbon intensity of construction materials (CI) (Eq. (1)) and material intensity (MI) for the local GDP (Eq. (2)).

(1) $$MI=DMI_{con}/GDP\;,$$

(2) $$CI=DMI_{con}\times Emissions_{con}\;,$$

To calculate the emission savings potential for Nagpur and Rajkot a median value for the carbon intensity of construction materials (CI) was first obtained from the cities of Bangalore and Mumbai. We then used local GDP values for Nagpur [43] and Rajkot [44] for the years 2014 and 2018 respectively and adjusted these via the national average growth rate [43], to establish the local GDP for 2020. Finally, multiplying the 2020 GDP values for Nagpur and Rajkot with material intensity correlated with GDP, the total amount of construction materials (DMI_con Rajkot/Nagpur_) was calculated (Eq. (3)).

(3) $$DMI_{conRajkot/Nagpur}=MI_{GDPRajkot/Nagpur}\times GDP_{Rajkot/Nagpur}\;,$$

After that the correction factors were applied (Table A.4). In a final step, construction material amounts were multiplied with the median carbon intensities to establish the GHG emission values for each scenario and the emission saving potential compared to the baseline (Eq. (4)).

(4) $$Emissions=DMI_{conRajkot/Nagpur}\times CI_{median}\;,$$

**Deriving material amounts embodied in the building stock via floor area and average materials used (the Philippines)**

As with the Indian cities, detailed data on the housing stock was also lacking for the Philippine cities. The approach taken and validated for the Philippine cities of Santa Rosa and Pasig extrapolated the total amounts of materials used in buildings by multiplying total floor area (m^2^) estimates with the amount and type of construction materials used per m^2^ (tons material/m^2^ of floor area). In this approach the total floor is calculated by multiplying the number of existing households in a specific year (2015) [45] by the average area of a residential unit the Philippines [46]. Quantities per type of construction material was derived from a life cycle analysis of structural systems of residential housing units in the Philippines [47]. Materials considered in the study include cement, sand, gravel and steel. Each material is given in kg and referenced for a 60 m^2^ house unit. The amount of each material is provided for four construction typologies with different structural designs. The average of the four types was calculated for the whole unit (60 m^2^) and then expressed per m^2^ unit.

**Material composition in the building stock for all case-study cities**

In the case of building material composition in Essen and Leipzig, the dataset shows a difference between single-family (SFH) and multi-family houses (MFH). Single-family houses in Essen, consist of an average of 309 t of building materials [40]. The majority of this is accounted for by concrete, other mineral materials and bricks. Sawn timber has only a small share, which continues to decrease slightly in the more recent age classes. In Essen for MFH, an average of 1272 t is used for a building [40]. The trends described for SFH also apply to MFH, except that they are more pronounced. Concrete consumption rises sharply until 1990 and then reduces again in the more recent age classes. Leipzig differs from Essen, especially in the 1979-1990 age class [40]. Concrete consumption is increased by 30%. Concrete dominates material consumption and accounts for 81% of total material consumption in this age class (Essen 53%) [40]. Wood also has a small share of total consumption in Leipzig, which is, however, even lower compared to Essen. As already described, the material compositions in India refer to Mumbai and Bangalore, therefore no compositions can be given for Rajkot and Nagpur. The construction of an average building in Bangalore consumes 16.7 tons of building materials, which is the lowest of all cities. Stone, steel and wood are the most commonly used materials in Bangalore. In Mumbai, it is concrete, steel and sand. For the construction of an average building in the Philippine cities, 23,2 t of building materials are used, which is significantly less than in Essen, for example. In both cities, the consumption of sand and gravel clearly dominates over concrete and steel. A distinction between single and multi-family houses could not be made for the Indian and Philippine cities due to a lack of data.

#### 2.3.2 Transport

An approach for all 6 cities was adopted to quantify the impact of mitigation measures for transport, with differences in the methodology being the result of data quality and availability, especially in relation to average travel distances.

**General approach to calculating transport emissions (good data availability)**

For Leipzig and Essen, household surveys and statistical data from the traffic departments were used, it contains the necessary information on individual traffic activity [48], [49]. Data for the Indian cities were extracted from the Comprehensive Mobility Plan for Nagpur [50] and the Low-Carbon Comprehensive Mobility Plan for Rajkot [34]. Both plans provided sufficient data to calculate average GHG emissions of passenger transport per year. Data availability for the Indian (Nagpur, Rajkot) and German cities (Leipzig and Essen) was sufficiently good to conduct the following generic approach without significant constraints. First we multiplied the modal split distribution with the corresponding trip lengths and the emission factor of the respective means of transport (see SI). Subsequently we multiplied average passenger transport emissions per trip by the total number of daily trips and extrapolated this figure to an entire year. In doing so, we were able to estimate the average annual GHG emissions of passenger transport in all six cities. This approach is similar to the approach of classical accounting protocols such as the IPCC or the German BISKO.

**Downscaling of transport data to calculate the baseline (low data availability)**

Detailed traffic indicators were lacking for the Philippine cities. Since Pasig is located in the metro Manila region and Santa Rosa is located close to it, values from the metro Manila region were used, which were then scaled down with local demographic data. For the Philippine cities of Pasig City and Santa Rosa daily GHG emissions were calculated by multiplying the emission factors of each transport mode with i) the share of each transportation mode [51], ii) the average distance of each trip (based on a model city, due to data unavailability) and iii) the number of daily trips for Metro Manila [51] and surrounding areas, after downscaling it with city population data [52]. A reference value of daily trips for Pasig City was found in a draft study [53] and the validity of the estimates was thereby confirmed.

**Transport activity for all case-study cities**

In Leipzig, the car (driver and passenger only) has the highest modal share with 36.5% [48]. Active transport, consisting of walking, cycling and bicycle- rickshaw, also has a high share with 46%. Public transport has a similar modal share (17.5%) as the bicycle with 18.7%. On average, 3.6 trips/person/day are made, each with an average length of 6.4 km.

In Essen, the car has an even higher modal share (54%) than in Leipzig. Active transport has a lower share of 25% compared to Leipzig [49]. However, the modal share of local public transport at 19% does not differ considerably from Leipzig. With 3.6 trips/person/day, just as many trips are made as in Leipzig, but with 8.7 km/trip they are longer.

The car has only a small share of 5.7% in Nagpur's transport system [50]. Two-wheelers have the largest share with 42.6%, followed by auto rickshaws with 19.8%. Active transport (walking and cycling) is less pronounced than in German cities at 15.9%. Public transport consists mainly of different types of buses (school bus, minibus, regular bus, chartered bus) and also characterizes the transport network with 15.5%. On average, the population in Nagpur travels 1.3 trips/person/day with an average length of 9.3 km.

In the less populous and smaller city of Rajkot, two-wheelers also have a high modal share (35%) [34]. However, walking is the most used form of mobility at 38%. The car (2%) and the bus (3%) do not have large modal shares. At 1.4 trips/person/day, they have a similar value to Nagpur, but these are significantly shorter at 3.5 km (Nagpur 7.6 km).

As with Rajkot, walking is the most used form of mobility in the Philippine city of Pasig city (31%), followed by jeepneys (19%) and tricycles (16%) [51]. Cars, as with Indian cities, have a smaller share with 8%. Bus and train together account for 11% of the trips. The average number of trips is 3 trips/person/day, with an average length of 9.7 km.

#### 2.3.3 Redensification

To estimate the impact of land use change and in particular redensification on emissions, we applied an urban scaling approach to redensification. Studies show that urban GHG emissions are roughly proportional to urban population or area [54], which describes the empirical scaling relationships between area, population and emissions. An extension of this is the Cobb-Douglas relationship, which is one of the best-known production functions. It can be used to analyse the effects of different populations (P) or areas (A) on emissions (C), both being related by density [54]:

(5) $$C=c_{1}P^{\beta_{P}}A^{\beta_{A}}\;,$$

$\beta_{P}$, $\beta_{A}$, and $c_{1}$ are obtained from fitting to the data with multi-linear regression models. Accordingly, Eq. (5) represents a generalisation, implicitly containing the density [54]. For the sample cities in the Global South, data from 68 cities on the surface area, emissions and population were used. GHG data with a consistent calculation methodology was required for the analysis, which was obtained from an ICLEI report [55] that contains all needed data from the year 2009 for cities in India. In addition, data from the carbon disclosure project for Philippine cities were analysed. Both use a very similar methodology of carbon accounting, which is based on the Greenhouse Gas Protocol. Since many cities do not account for scope 3 emissions and were therefore not available, only scope 1 and 2 emissions were considered. The analysis of such large cohort of cities was used to calibrate the model for calculating the parameters.

To conduct the analysis for Essen and Leipzig, 2018 data from 47 German cities was taken from the Climate ActionPlanner, an internet-based software for balancing greenhouse gases [56]. It actively implements “Bilanzierungs-Systematik Kommunal” (BISKO), which is the most widely used accounting tool at the municipal level in Germany, including Scope 1 and 2 emissions.

After determining the parameters, two different scenarios were analysed with the Cobb-Douglas function, in which the area and the population were changed. Therefore Eq. (5) was changed for this Eq. (6). The aim was to find out the influence of increased urban density on C, where A is lower than P. In order to accurately account for the development dynamics of individual cities, city-specific scenarios were defined.

(6) $$C=\log c_{1}+\beta_{P}(\log x_{P}+\log P)+\beta_{A}(\log x_{A}+\log A),$$

According to the allometry of P and A, the increase in A and P differed by a factor of 0.8 [57]. Recognising the correlation of population increase and settlement area expansion ($x_{a}$) (allometry of P and A), the first scenario (S1) factored in estimated city-specific percentage population growth ($x_{p}$) figures up to 2030, by increasing P by this value in the function. Here, this scenario was supposed to represent urban sprawl due to the increase in P and A. The second scenario (S2) represents a redensification, where P was increased by the same value in the function as for the 2nd scenario, but A was held constant (same value as in the first scenario) to explore the impact of population growth without urban expansion. The baseline scenario represented the status quo and no change in P and A. The German cities have the lowest population growth, followed by the Indian cities and the Philippine cities with the highest population growth.

Subsequently, the emissions (C) of the Cobb-Douglas function could be compared with each other and the additional GHG savings of the 3rd scenario compared to the 2nd scenario could be examined.

## 3 Results

In the section following, the impacts of the selected mitigation measures are described according to the field of action.

### 3.1 Mitigation potential sustainable building

The what-if analysis in sustainable construction involved calculating two scenarios to quantify the emission saving potential of timber in construction: substitution 5- and 15-% of construction material with timber.

**Figure 2:**
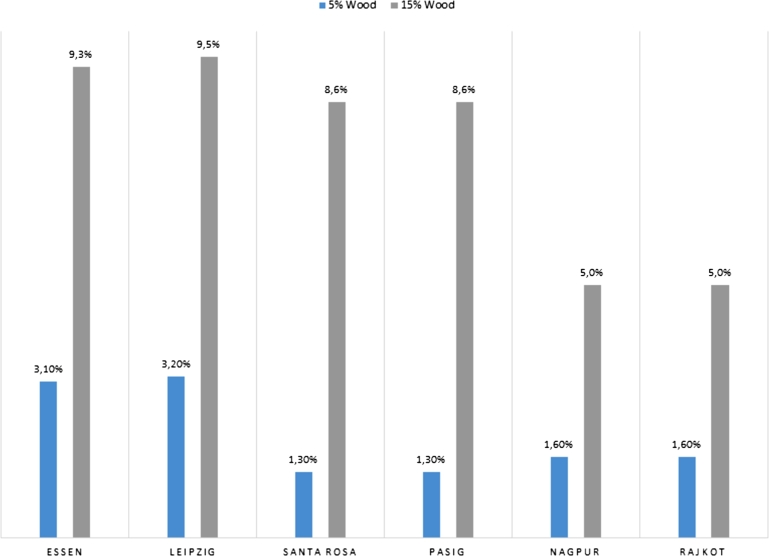
Emission saving potential under the two scenarios for all cities in the construction [%]. The what-if analysis in sustainable construction involved calculating two scenarios, a 5% (left bars) and 15% (right bars) intensification of wood as a building material. All percentage emission savings refer to the status quo of the calculated building material emissions and relate to the latest available data.

With a 5% increase in the use of wood as a building material, the potential savings range between 1.6 and 3.2% (Fig. 2). The lowest GHG savings are achieved in the Philippine Cities, with only minor differences between them. Indian cities have only slightly higher potential (1.6%). Essen with 3.1% has similar mitigation potentials than Leipzig with 3.2%, which is also the highest value in the first scenario.

With a 15% increase in the substitution of conventional building materials with sustainably managed wood, the GHG savings range between 5 and 9.5% (Fig. 2). The two Indian cities have increased the GHG savings with 5%, whereby the two cities have the lowest savings potentials of the six. The Philippine cities each have 8.6% emission savings. Essen, with 9.3%, again has slightly lower savings than Leipzig with 9.5%.

### 3.2 Mitigation potential of the transport sector

**Figure 3:**
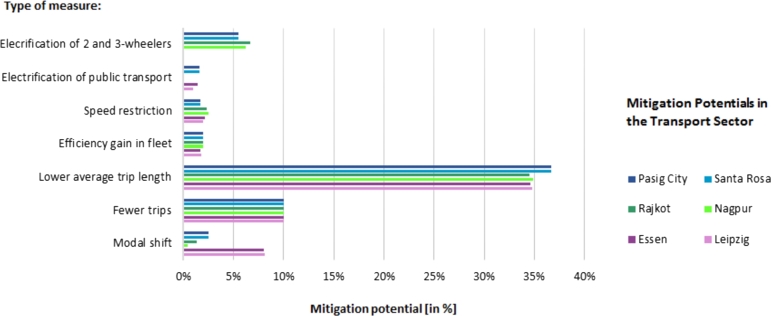
Mitigation potentials for all cities in the transport sector. All percentage emission savings refer to the status quo of the calculated transport emissions and relate to the latest available transport data.

The measure to reduce average travel length has the highest mitigation potential emissions savings compared to the baseline (Fig. 3). Rajkot has the lowest saving potential of the cities at 34.5% and the Philippine cities have the highest potential at 36.7%, with the Philippine cities also having the highest average distances traveled. This measure is followed by the fewer trips with 10%, whereby the cities do not differ in relative terms (Fig. 3). With a modal shift from cars to non-motorised transport, the German cities of Leipzig and Essen also have the highest mitigation potentials in this action field with 8% emission savings based on their high share of cars. In Nagpur, a modal shift has a potential of only 0.4% and in the Philippine cities of 2%. On the other hand, electrification of two and three wheelers would have an effect mainly in the Indian and Philippine cities (Rajkot 6,7%, Nagpur 6,2%; Philippine cities: 5,5%). Despite an electricity grid with a low share of renewables, Indian cities show the highest emission savings in 2- and 3-wheeler electrification, at 6.7% (Rajkot) and 6.2% (Nagpur). When the public bus fleet in Indian cities is electrified, it leads to an increase in emissions of 0.1 to 0.3%. Leipzig would have reduced emissions the most with 4%, followed by the Philippine cities with 2% and Essen with 1.4%.

### 3.3 Mitigation potential of the redensification measures

The parameters of Eq. (5) differed substantially between the German and the Asian data sets (Table 2). $\beta_{A}$ was higher by a factor of 10 in the German cities than in the Indian cities. Since $\beta_{A}$ is close to 0 in the Asian cities, difference to conventional urban scaling is minor and has minor impact on GHG emissions. Furthermore, $\beta_{P}$ is near zero in Indian cities and thus linear with GHG emissions. The Eq. (7) shows the linear regression model for the German cities and the correlation of the emissions C with the $c_{1}$ introduced in Eq. (5), the population P with the corresponding $\beta_{P}$ and the area A and the corresponding $\beta_{A}$, as reported in Table 2. It is a variation of Eq. (6), excluding growth values.

(7) C=3.1081+0.8675log⁡P+0.0994log⁡A,

For all cities, emissions increase in scenarios S1 and S2, though emissions increase at a slightly slower rate in S2. Asian cities have the higher percentage increase from the status quo to S1 given the higher population growth rates in S1 compared to current emissions.

**Table 2:** Parameter are obtained from fitting to the data with multi-linear regression models.

Parameter	Estimate
*β*_*P*_ asian	0.9189
*β*_*P*_ german	0.8675
*β*_*A*_ asian	0.0183
*β*_*A*_ german	0.0994
*c*_1_ asian	1.1542
*c*_1_ german	3,1081

If population increases (S1), the city increases GHG emissions (Table 3). However if, as in S2, P increases and A remains constant, urban density increases and emissions are reduced compared to the joint increase of P and A in S1 (representing urban sprawl).

**Table 3:** Results of the different Cobb-Douglas function depending on the city and scenario: Actual is the baseline, S1 represents urban sprawl due to the increase in P and A, S2 represents a redensification, where P was increased and A was held constant.

City	Scenario	Population Growth [%]	Increase Area [%]	Emissions [tCO2]	GHG Change compared to actual and S2 vs. S1[%]
Leipzig	S1	7	5,6	4.214.307	6,21%
	S2	7	0	4.191.539	-0,54%
Essen	S1	5	4	4.007.563	4,52%
	S2	5	0	3.991.966	-0,39%
Nagpur	S1	24	19,2	3.164.981	18,20%
	S2	24	0	3.154.808	-0,32%
Rajkot	S1	36	28,8	1.961.681	24,96%
	S2	36	0	1.952.602	-0,46%
Pasig City	S1	56	44,8	1.291.097	34,00%
	S2	56	0	1.282.366	-0,68%
Santa Rosa	S1	41	32,8	484.292	27,45%
	S2	41	0	481.780	-0,52%

This expectation is confirmed by our results: the emission savings from S2 to S1 differ between the German and Asian cities, but are all in a similar range of 0.39-0.68%, despite the growth rates of P and A are significantly higher in the Asian cities (Table 3). Although at a low level, Pasig City has had the highest savings in S2 also due to the highest growth rates. Relative to the increases in P and A, the German cities have the higher savings compared S2 to S1, which is related to the low Asian $\beta_{A}$ parameter. Nevertheless, the savings achieved in the second scenario are also small in relation to the savings in the other fields of action.

## 4 Discussion

The emissions of the building stock materials are currently omitted from common GHG-inventory calculation protocols (e.g., IPCC guidelines, GPC and the German BISKO). We derive indicators that enable general recommendations for action in climate-oriented urban planning. Approaches and recommendations are often evaluated on the basis of homogeneous data, yet in reality, city-level data quality and availability varies substantially across the world. While several studies examine how spatial form and GHG emissions are related to individual emission sectors they do not take the data heterogeneity between cities into account [26], [58]. To map the data heterogeneity, we have tried to adapt frequently used approaches such as material flow analysis or the calculation of transport emissions according to classical accounting protocols to the data availability and to combine them with specific approaches. Therefore, in our work we have developed several methodological approaches for a city-specific analysis, thus providing users with quantification approaches that are applicable across cities.

### 4.1 Discussion mitigation potentials

With the expected expansion of the infrastructure, attention must be paid to the way in which new building infrastructure is constructed. The substitution of conventional building materials with wood was analyzed as having the highest mitigation potential. The mitigation potential depends on the material distribution in the base scenario. The share of steel and ferrous metals for Indian cities (18%) is higher compared to German cities (4%) or Philippine cities (5%). Deploying the correction factor (4 tons of wood substitute 1 ton of steel) cause an increase in material consumption for the Indian cities but a decrease for the other cities. Therefore, the mitigation potential for India is on average lower. As part of the 100 climate-neutral and smart cities by 2030, Leipzig must implement very ambitious measures. The very challenging substitution of 15% of the entire building stock would halve Leipzig's total emissions and come closer to the step of climate neutrality.

The relevance of the building sector for climate change mitigation has also been identified by Guo et al. (2017), where they estimated that the energy consumption and carbon emissions of cross-laminated timber (CLT) buildings are 10% and 13% lower than those of reinforced concrete buildings, respectively, based on a full life cycle assessment [59]. In the report by the International Resource Panel (IRP) report [60], a what-if analysis was also conducted with different measures for various cities, the highest emission saving also had the substitution of concrete based materials with bio-based materials, mainly CLT. With complete substitution by biobased materials, 64% GHG savings could be achieved. The differences compared to our study can be explained by the different scenarios applied. They also argued that mitigation potentials in bioresource rich regions could be even higher. Especially in countries of the global south, other traditional, bio-based materials, e.g. bamboo, straw, hemp, must also be taken into account in the transformation towards sustainable construction [37]. Studies showed that substituting traditional steel roofs with low-cost, alternative building materials, such as tiles made from fly ash led to energy savings of more than 20% for the materials used [60], [61]. The pure use of wood as a substitute material could lead to land use and target conflicts, which is why the additional use of alternative sustainable materials is necessary [62].

The multimodal transport measures showed varying GHG impacts. If Leipzig as one of the 100 climate-neutral cities would adopt all the transport-specific measures considered in this study, they would halve their transport emissions. Both German Cities Leipzig and Essen, show a significant share of individual transport via cars, therefore a modal shift to fewer cars leads to higher mitigation potential, compared to the other cities. Creutzig et al. 2012 also showed significant GHG savings with a modal shift from car to less emission-intensive transport modes. Due to the correlation of polycentrism and traffic reductions, the modal shift effects can also be enhanced by considering polycentric structures in new districts [58]. In contrast to Germany, modal split in fast developing cities is currently changing rapidly in favour of individual mobility, mainly cars: For example, the number of registered cars in the city of Nagpur increased between 2014 and 2017 by 71%, whereas the population increased by 10%. For the Indian cities, the high modal share of 2- and 3-wheelers, is even expected to increase in the future [63]. Taking into account this trend of increasing car ownerships, there is a risk to further strengthen the path dependency towards a car-oriented city [64]. It is assumed that the implementation of different groups of measures in the early development phases of infrastructure can prevent the dominance of the car [65], which highlights the need to take urban dynamics into account for planning processes. Furthermore Bongardt (2010) showed that the potential of avoidance of trips in the Global South are twice as effective as those that can be achieved through modal shifts or technological improvements.

The mitigation potential of public transport electrification depends strongly on the current share of electrified public transport and the share of renewable energy in the electricity grid [66]. The high share of coal in India's electricity grid even led to an increase in emissions in the electrification scenario. Given the higher share of renewables in the electricity grid, the Philippine and German cities have higher mitigation potentials. Particularly in the transport sector, however, it is more effective to aim for a mix of measures: like modal shift, avoidance of trips, electrification and also compact densification measures, rather than concentrating on a single measure [67].

The results for redensification in already densely populated cities indicate a low influence of the area on GHG emissions ($\beta_{A}$ barely correlates with C). The difference in emission savings between Asian and German cities is mainly due different parameters ($\beta_{P}$ correlates almost linearly with C). Compared to the German cities, the Asian cities had a $\beta_{A}$ that was five times lower. This means that cities in the Global South are characterized by high urban densities and the associated more efficient use of land [68]. The already high urban density then leads to lower greenhouse gas emissions per capita. This finding is in line with similar analyses for US cities: Cities in the US have low densities and the concept of the car-oriented city is widespread there [69]. The analyse for these showed significantly higher values for $\beta_{A}$ compared to our parameters [54]. Given the low $\beta_{A}$, the change in area also did not have a strong impact on the emission saving potentials. Especially when compared to the sectoral potentials of the other two fields of action, which were significantly higher. Few studies have carried out similar quantitative scaling analyses with urban density, Ramaswani et al.,2012 examined, albeit without a scaling approach, the impact of doubling density in the urban core in Minneapolis, which affected 3.7% of the total population. A doubling resulted in a 1% reduction in vehicle miles traveled. Our study directly calculated GHG reductions and only drew a qualitative link to transportation.

In our case scenario of a direct increase in urban density, the resulting impacts on other systems must also be taken into account in addition to the mitigation potentials. The tension between redensification and the prevention of the urban heat island effect is a typical example of trade-offs, which can only be resolved by integrated design solutions, and/such as nature-based solutions [70], [71].

### 4.2 Limitations

The objective of the work was to analyse mitigation potentials despite a heterogeneous data availability across cities for different fields of action. Some simplifications and assumptions had to be made.

For the building sector, we quantified the effect of replacing large amounts of construction material with timber. We assume that the emissions of a forest caused by forest management and timber harvesting are net zero, this implies that the timber originates from regional and sustainably managed forest [72]. For a holistic potential analysis of wood substitution in the construction sector, the entire life cycle from the changes of carbon storage capacity of the forests to the CO_2_ storage function in buildings would need to be considered. In the case of long - term wood removal from forests, the actual mitigation potential for substitution is lower than indicated in this study [73]. Another point that has not been taken into account is the end of life stage of wood products. Often wood products are burned at the end of their life stage to produce energy, which reduces their mitigation effects [74], [75].

In addition, for simplicity, the carbon intensities for all materials are average mean values that neglect the large variability in building types and construction materials and not country or city specific characteristics [76]. This is also reflected in the calculation of material consumption for India and the Philippines. For the Indian cities, a top-down approach was chosen, which did not allow for a differentiation of the cities and a disaggregated analysis. For the Philippines, differentiated bottom-up data for current wood consumption was lacking.

Data availability also affected the approaches for the transport sector: Only travel distance data could be collected for the Metro Manila region, hence both cities do not differ in their potential. The results on redensification showed high standard residual errors (0.23), indicating a high level of “noise” in the data sets used. However, with the Cobb-Douglas function, these have a considerable influence on the results and thus also on the emission savings.

In some cases, older data was used, which is an issue that many cities face. Efforts have been made to use the most recently published data, which are similar for all cities. However, due to the nature of the scenarios, the timing of the data is less important. In addition, mitigation pathways on time scales of several decades represent a commitment and depend less on current data availability and more on future assumptions about change. An example is the case of the redensification approach, where the data is old but was not crucial for the success of the approach. The aim was primarily to use statistical approaches to determine the correlation and, based on this, to derive the relative results.

All approaches were performed assuming a what - if - analysis, disregarding dynamic developments, such as demographic, economic or political changes. Further research is needed on the consideration of economies such dynamics over time, which would enable further detailed statements on the interrelationships between urban infrastructures and emissions.

### 4.3 Conclusions

In conclusion, the research reveals that successful urban interventions directed towards a climate-oriented development are place-specific, time-bound and highly dependent on the status quo with regard to the use of building materials, modes of transportation and the state of the urban form. Substitution of conventional building materials (concrete and metals) with wood carries the highest emission savings potential among the fields of action studied across all sample cities. However, to avoid trade-offs, e.g. increased land-use competition, wood-based construction materials need to be enhanced in terms of material properties (e.g. CLT) and complemented with other biogenic materials.

For the urban form, the research revealed a limited impact of population density (of the area) on emission reductions assuming an increasing population with maximized reduction potential achieved through a reduction in land area with steady population. Considering trade-offs with climate adaptation goals (e.g. avoidance of heat island effect, vulnerability to flooding), the results also suggest that interconnected polycentric urban systems can achieve co-benefits and synergies between climate change mitigation and adaptation. Consequently, the focus of climate-oriented urban development needs to be on the urban form with the objective of facilitating a more polycentric form, exploiting efficiency gains from population density and reduced transport needs as well as enhancing urban resilience.

This research also contributes to advancing mixed quantification approaches for assessing the carbon reduction potential with a focus on practicability and replicability for local practitioners and authorities. Despite different data availability, it was possible to derive applicable calculation approaches for all cities. This allows cities to incorporate mitigation potentials in different fields of action, which have not been considered so far, into their mitigation pathway. It demonstrates that with mixed bottom-up and top-down approaches for handling data heterogeneity, plausible results can be achieved that can inform policies on the ground for climate-oriented urban development. However, more work is needed to further advance mixed approaches and make them operational for local authorities.

A considerable expansion of urban infrastructure can be expected in the future, especially in the Global South. However, the increase in new urban areas also offers an opportunity to design for an appropriate functional density, thus avoiding path dependencies or lock-ins with negative consequences such as urban sprawl, functional segregation and high traffic volumes [77]. In existing urban areas, redensification potentials vary according to the respective local context and are often more difficult to exploit. Further research is needed, especially with regards to the impacts of sector competition as well as robust demand supply estimates of “timber cities”, local emission factors and local building design solutions. Furthermore, a deepened quantitative study on the synergies between redensification and transport could further reveal empirical results that can inform policies at different levels.

## CRediT authorship contribution statement

Fabian Reitemeyer, David Fritz, Nikolai Jakobi, Juergen P. Kropp, León Díaz-Bone and Carla Mariño Viteri conceived and designed the experiments. Fabian Reitemeyer, David Fritz, Nikolai Jakobi and Carla Mariño Viteri performed the experiments. Fabian Reitemeyer, David Fritz, Nikolai Jakobi, Carla Mariño Viteri and Juergen P. Kropp analyzed and interpreted the data. Fabian Reitemeyer, David Fritz, Nikolai Jakobi, Carla Mariño Viteri and León Díaz-Bone contributed reagants, materials, analysis tools or data. Fabian Reitemeyer, Nikolai Jakobi, Juergen P. Kropp and León Díaz-Bone wrote the paper.

## Data availability statement

Data will be made available on request.

## Declaration of Competing Interest

The authors declare that they have no known competing financial interests or personal relationships that could have appeared to influence the work reported in this paper.

## Figures and Tables

**Table A.4:** Correction factors to convert the amount of wood to a corresponding amount of abiotic material were derived, which consider different wall thicknesses, weights and densities.

Materials	Correction factor
Concrete	4.42
Brick	2.56
Other minerals	2.50
Synthetic materials	0.50
Metals	0.25

**Table A.5:** Direct and indirect fuel life cycle emission factors from GEMIS and ecoinvent database.

Fuel type	Direct	Indirect	Total	Unit
Gasoline	2.30	0.35	2.65	kg GHG/l
Diesel	2.71	0.41	3.12	kg GHG/l
Gas	1.69	0.34	2.03	kg GHG/kg
Electricity India	-	-	0,87	kg GHG/kWh
Electricity Philippines	-	-	0,65	kg GHG/kWh

**Table A.6:** Direct and indirect building material life cycle emission factors from GEMIS and ecoinvent database.

Materials	Emission factor	Unit
Concrete	0.177	kg CO2e/kg
Brick	0,2569	kg CO2e/kg
Other minerals	0,3873	kg CO2e/kg
Sawn timber/processed timber	0,2872	kg CO2e/kg
Other regrowth	0,1436	kg CO2e/kg
Synthetics	3,09	kg CO2e/kg
Metal, ferrous	1,344	kg CO2e/kg
Bitumen	0,417	kg CO2e/kg

**Table A.7:** Average annually GHG emissions of passenger transport per person in tons CO2e for all cities.

Essen	Leipzig	Santa Rosa	Pasig	Nagpur	Rajkot
1.3	0.581	0.25	0.25	0.15	0.05

## Appendix A

**Correction factors used for the sustainable building sector**

Braun et al., assessed in 2016 the GHG mitigation potential from forests and wood use in Austria. One of the research question was to calculate the emissions avoided when using HWP (harvested wood products) instead of abiotic materials. A simple substitution per mass, volume or density is not possible considering real application; i.e. 1 $m^{3}$ concrete is not likely substituted by 1 $m^{3}$ of hard wood. We use the same correction factor to convert the amount of abiotic materials to wooden based materials, with some simplification (Table A.4)

**Emission factors used**

Emission Factors for all materials in the building sector (Table A.5) as well as for the mobility sector (Table A.6) were used from the ecoinvent database and the GEMIS Software. Where available, region specific data (Table A.7) were used. The GEMIS model (Global Emission Model for Integrated Systems) is a computer-based tool that calculates the climate impacts of different systems and processes in a simple, precise and above all comprehensive way and relates them to each other.

GEMIS takes account of all major processes ranging from primary energy and raw material production to the supply of useful energy and materials, including e.g. auxiliary power and material demand for the building of energy installations and transport systems, and thus makes it possible to account for process emissions, along with the direct emissions.

These emissions factors include the direct emissions as well as the indirect emission, life cycle emissions factors. This is necessary to address possible trade-offs between ecological burdens, f.e. within the mobility sector. Here, a switch to electric mobility may decrease direct emissions of the vehicles to zero, but the propulsion energy is electricity and therefore the indirect emissions of the vehicles need to be considered. Specific consumption data (source: Low - carbon Comprehensive Mobility Plan) and the fuel mix derived from the Low - carbon Comprehensive Mobility Plan.
